# Supplementary material for: MetaRibo-Seq measures translation in microbiomes
Source: Nat Commun. 2020 Jun 29;11:3268. doi: 10.1038/s41467-020-17081-z (PMC7324362; doi:10.1038/s41467-020-17081-z)
Supplement: Supplementary file 10 — Supplementary Data 7 [file 41467_2020_17081_MOESM10_ESM.zip › File2/Confidence_High_Taxonomy/29808_out.krona.html]

Javascript must be enabled to view this page.

members
magnitude
magnitudeUnassigned
count
unassigned
taxon
rank

29808\_out

76

superkingdom
76
2

1239
phylum
76

76
class
186801


SRS075984\_contig\_number\_contig-100\_999.1000
order
1
186802
76

186803
family
71

71
species

SRS011084\_contig\_number\_38016SRS013965\_contig\_number\_17442SRS014313\_contig\_number\_contig-100\_541.53122SRS014613\_contig\_number\_contig-100\_763.120751SRS014736\_contig\_number\_contig-100\_345.72158SRS014948\_contig\_number\_24843SRS015217\_contig\_number\_13724SRS016018\_contig\_number\_3733SRS016056\_contig\_number\_contig-100\_6728.6728SRS016495\_contig\_number\_749SRS017191\_contig\_number\_17695SRS018427\_contig\_number\_contig-100\_353.186517SRS018623\_contig\_number\_contig-100\_178.132307SRS019267\_contig\_number\_contig-100\_736.61577SRS022137\_contig\_number\_22153SRS023346\_contig\_number\_contig-100\_2366.58350SRS042628\_contig\_number\_24228SRS043701\_contig\_number\_17894SRS044535\_contig\_number\_contig-100\_442.147373SRS046717\_contig\_number\_11098SRS047044\_contig\_number\_18743SRS047433\_contig\_number\_9672SRS048060\_contig\_number\_13674SRS048870\_contig\_number\_contig-100\_420.136362SRS049446\_contig\_number\_12984SRS049900\_contig\_number\_10970SRS050422\_contig\_number\_13535SRS050998\_contig\_number\_contig-100\_204.101893SRS051882\_contig\_number\_15345SRS053214\_contig\_number\_12509SRS053335\_contig\_number\_14842SRS055982\_contig\_number\_19862SRS058723\_contig\_number\_contig-100\_473.63708SRS063370\_contig\_number\_contig-100\_661.84786SRS064276\_contig\_number\_contig-100\_64.231138SRS075021\_contig\_number\_1803SRS076804\_contig\_number\_4596SRS077194\_contig\_number\_contig-100\_429.192449SRS077294\_contig\_number\_19294SRS077335\_contig\_number\_contig-100\_635.60340SRS077502\_contig\_number\_contig-100\_722.104318SRS098073\_contig\_number\_16483SRS098644\_contig\_number\_48429SRS1041036\_contig\_number\_1080SRS1041090\_contig\_number\_1425SRS104197\_contig\_number\_contig-100\_886.3936SRS104311\_contig\_number\_contig-100\_650.266627SRS104327\_contig\_number\_7803SRS104400\_contig\_number\_contig-100\_311.341345SRS104485\_contig\_number\_contig-100\_544.44349SRS104975\_contig\_number\_1455SRS1055049\_contig\_number\_contig-100\_230.97398SRS1055056\_contig\_number\_4783SRS142503\_contig\_number\_contig-100\_475.332069SRS143342\_contig\_number\_19948SRS143466\_contig\_number\_24332SRS143991\_contig\_number\_contig-100\_2549.118567SRS144362\_contig\_number\_27796SRS146764\_contig\_number\_28805SRS147272\_contig\_number\_contig-100\_4572.4573SRS147346\_contig\_number\_contig-100\_119.355931SRS147614\_contig\_number\_10125SRS147766\_contig\_number\_7670SRS148196\_contig\_number\_41977SRS148253\_contig\_number\_contig-100\_468.100291SRS148424\_contig\_number\_27169SRS149325\_contig\_number\_14313SRS893252\_contig\_number\_contig-100\_445.118098SRS893295\_contig\_number\_10248SRS893341\_contig\_number\_11068SRS893369\_contig\_number\_6455
39491

186806
family
4

4
genus
1730

species
3
1897003

SRS019397\_contig\_number\_12209SRS042284\_contig\_number\_2447SRS893279\_contig\_number\_4842

1
species
142586

SRS1041144\_contig\_number\_5161
